# Sugar Shockwaves: How the Fructose–Glucose–ChREBP Pathway Hijacks Liver Metabolism

**DOI:** 10.3390/life16081313

**Published:** 2026-08-11

**Authors:** Alejandro Gugliucci

**Affiliations:** Department of Research, Touro University California, Vallejo, CA 94592, USA; alejandro.gugliucci@gmail.com

**Keywords:** atherosclerosis, ChREBP, DNL, fructose, insulin resistance, MASLD, methylglyoxal, MetS, obesity, triglyceride-rich lipoproteins

## Abstract

Recent research establishes carbohydrate response element-binding protein (ChREBP) as a central regulator of fructose-induced hepatic lipogenesis, underscoring its critical role in mediating the effects of excessive sugar intake on liver metabolism. Consequently, this narrative review presents a perspective of hepatic sugar metabolism and ChREBP signaling, highlighting areas for further exploration to better understand and address the metabolic consequences of fructose-driven activation of ChREBP. The discussion encompasses five principal domains: (1) fructose metabolism; (2) the structure and function of ChREBP; (3) interactions between glucose and fructose metabolism with ChREBP activity; (4) mechanisms that intensify fructose’s influence on ChREBP pathways; and (5) future research opportunities. Under physiological conditions, ChREBP mitigates accumulation of glycolytic intermediates; however, excessive sugar consumption overwhelms these capacities, thereby increasing lipogenesis. Fructose demonstrates notably stronger activation of ChREBP in vivo relative to glucose. Given ChREBP’s dual role in maintaining metabolic homeostasis and promoting insulin resistance under high fructose intake, therapeutic targeting poses considerable challenges. We propose the testable hypothesis that the MG generated from excessive fructose metabolism may bind to ChREBP’s lysine residues, stabilizing the protein and reducing degradation by impeding ubiquitination. This stabilization of ChREBP would prolong its activity, thereby further promoting sustained hepatic lipogenesis and exacerbating the risk of MASLD. Experimental and clinical research is needed to confirm its validity and define the molecular pathways involved. Future studies should aim to identify glucose metabolites involved in ChREBP regulation and clarify its beneficial versus adverse effects. The specific functions and regulation of ChREBPβ vs. ChREBPα remain to be elucidated.

## 1. Introduction

Metabolic syndrome (MetS) and its related consequences—including obesity, diabetes, and cardiometabolic disorders such as metabolic-associated fatty liver disease (MASLD)—are rising at a considerable rate, primarily due to intricate interactions among genetic predisposition, environmental factors, dietary habits, and physical activity [1,2,3]. The increased accessibility of sugar, especially through sweetened beverages containing high-fructose corn syrup (HFCS) or sucrose, coincides with the growing prevalence of these health conditions [4,5,6,7,8].

Beginning in the 1960s and further refined over the past 20 years, research has shown that increased fructose consumption correlates with swift rises in triglyceride concentrations and hyperinsulinemia in both human and animal studies [9,10,11,12,13,14]. Fructose significantly contributes to hepatic fat accumulation and dyslipidemia. Some controversy still exists as some meta-analyses contend that total caloric intake is the predominant determinant [15,16]. Public health organizations recommend that less than 10% (ideally under 5%) of daily energy comes from free sugars to reduce the risks of obesity and metabolic diseases [9,10,11,12,13,14]. Many regions exceed these guidelines, and added sugars, especially from sugar-sweetened beverages, are a major source of excess intake.

Dysregulation of lipid metabolism, particularly in the liver, includes de novo lipogenesis (DNL, the synthesis of fatty acids from acetyl-CoA), re-esterification of free fatty acids (FFA), and increased production of trioses attributed to excessive glycolytic and fructolytic pathways [17,18,19,20,21,22,23]. ChREBP functions as the principal glucose-responsive regulator of hepatic DNL, serving as a central mediator for hepatic TG synthesis [24,25,26,27,28]. Under normal conditions, liver cells prevent harmful buildup of glycolytic intermediates by promoting glycogen formation and exporting glucose through ChREBP activation [26,27,29,30,31,32]. However, excessive sugar intake or having poor glycemic control can overpower these protective mechanisms and result in unrestrained lipogenesis [25,31,33,34]. Whereas high-glucose concentrations robustly activate ChREBP in vitro, hepatic ChREBP activity in vivo appears more responsive to sugars other than glucose. Specifically, hepatic ChREBP is acutely and potently activated by fructose ingestion [31,35,36]. The stronger effect of fructose compared to glucose in activating ChREBP is likely due to the different metabolic pathways and enzymes involved in processing each sugar within the liver. In this context, interrupting the feedforward lipogenic pathway—particularly through modulation of ChREBP—may offer a compelling therapeutic approach for the treatment of MASLD and triglyceride dyslipidemia [30,32,37,38]. Conversely, decreasing dietary sugar intake to attenuate ChREBP expression may serve as an important preventive measure.

This narrative review provides a concise overview of the hepatic pathways involved in glucose and fructose metabolism, with a particular emphasis on the role of carbohydrate response element-binding protein (ChREBP) signaling.

The review discusses the metabolic processing of fructose in the liver and explores the structure and function of ChREBP. It outlines the connections between glucose, fructose, and ChREBP, emphasizing how these metabolic pathways interact within hepatic tissue.

Key areas for further research are highlighted, including the mechanisms underlying ChREBP activation and regulation in the context of carbohydrate metabolism.

The review also proposes a novel and testable hypothesis: excess methylglyoxal, a by-product produced during fructolysis, may serve as a stabilizing mechanism that prolongs the action of ChREBP.

## 2. Fructose: A Potent Activator of ChREBP

Increasing evidence indicates that consuming too much fructose is a contributor to MASLD and TRL dyslipidemia. When consumed above moderate levels, fructose overwhelms the small intestine’s absorption capacity, forcing the liver to metabolize the surplus, which directly increases fatty acid synthesis [17,39,40,41,42]. This metabolic overload is not limited to processed sugars; even “healthy” options like high-fructose corn syrup or apple juice can trigger fructose malabsorption, disrupting the gut microbiome and intestinal barrier.

### 2.1. Fructose Metabolism in the Liver: An Overview of Four Distinct Pathways

#### 2.1.1. Fructolysis Employs Three Unique Enzymes

**Figure 1:**
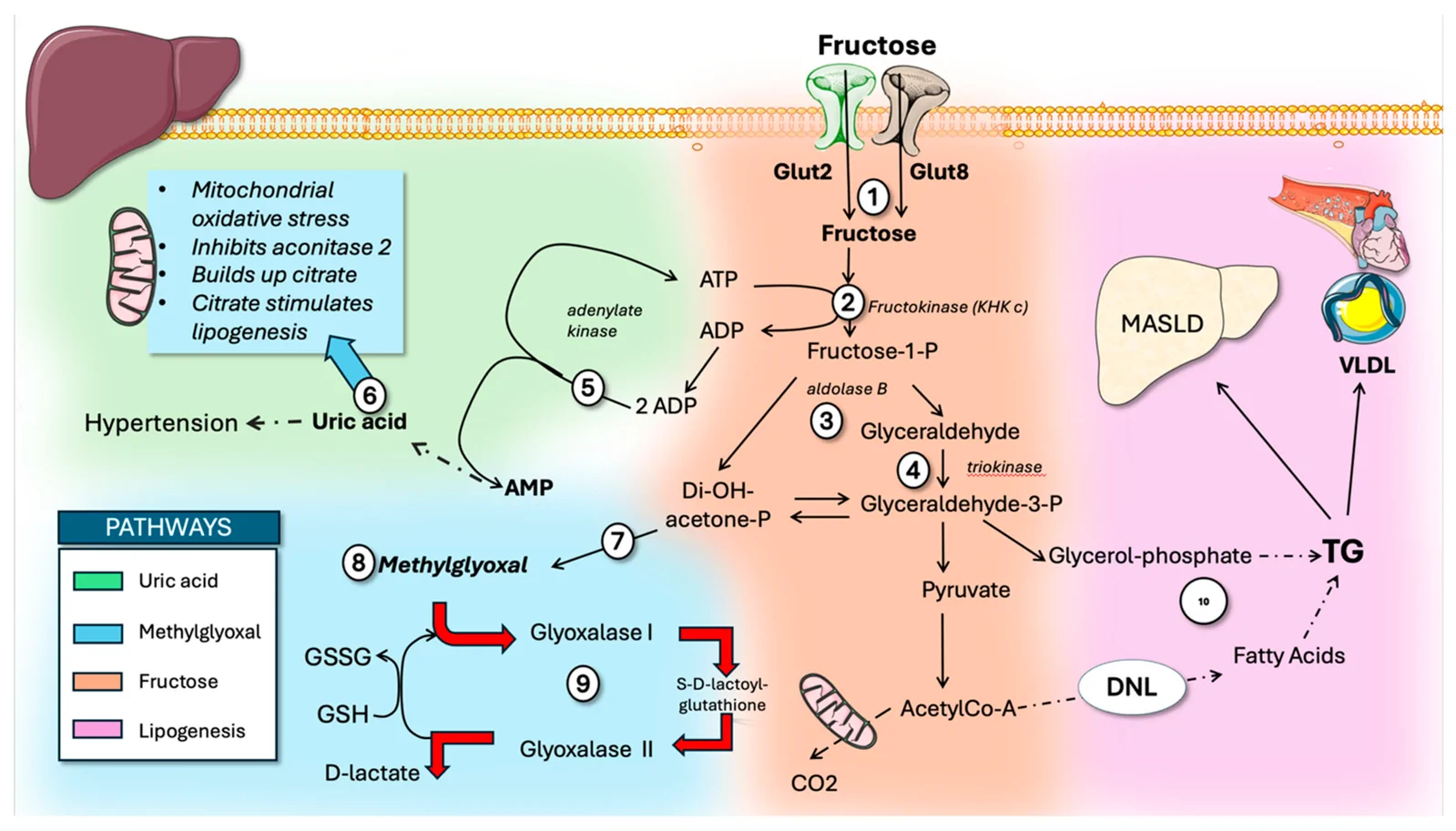
The four main pathways of fructose metabolism by the liver. Center panel, orange background shows the specific fructolytic pathway: (1) The liver takes up fructose via GLUT2 (and GLUT8) from the portal vein (center orange background). A set of three specialized enzymes drives hepatic metabolism of fructose, accounting for over 80% of the total fructose load. The rest of fructose metabolism shares the canonical enzymes for glycolysis. The three enzymes—(2), (3), and (4)—convert it into triose-phosphates, which integrate with those from glycolysis and gluconeogenesis. (2) The enzyme ketohexokinase c (KHKc), also called fructokinase, irreversibly phosphorylates fructose to fructose-1-phosphate—a step unique to fructolysis. Depending on metabolic conditions, these trioses are used to produce acetyl-CoA, triglycerides, glycogen, lactate, or fatty acids. (3) Next, F1P is split into two three-carbon molecules, dihydroxyacetone phosphate (DHAP) and glyceraldehyde, by aldolase B. (4) Glyceraldehyde is then phosphorylated by triokinase into glyceraldehyde 3-phosphate. Top left (green background) summarizes uric acid metabolism: (5) fructose phosphorylation leads to a reduction in free phosphate and increase in AMP, which in turn activates AMP deaminase and stimulates (6) uric acid synthesis. Uric acid plays a critical role in the development of hypertension, further enhances fructose metabolism, impairs mitochondrial function, and builds up citrate. Citrate is the main transporter of carbons in the form of acetate, as substrate for DNL in the cytosol. Bottom left (blue background): fructose metabolism (7) increases methylglyoxal (8), which is toxic and metabolized (9) by glyoxalases. Right panel (mauve background) summarizes de novo lipogenesis (DNL) using acetyl-CoA and (10) glycerol backbone for TG as well as its consequences: metabolic-associated steatotic liver disease (MASLD) and very-low-density lipoprotein (VLDL) dyslipidemia. The figures were partly generated using Servier Medical Art (Version 5), provided by Servier, licensed under a Creative Commons Attribution 3.0 unported license.

Figure 1 illustrates the primary pathway of fructolysis (center, orange). The liver takes up fructose via GLUT2 (and GLUT8) from the portal vein. A set of three specialized enzymes drives hepatic metabolism of fructose, accounting for over 80% of the total fructose load. The rest of fructose metabolism shares the canonical enzymes for glycolysis [19,20,22].

First, the enzyme ketohexokinase c (KHKc), also called fructokinase, irreversibly phosphorylates fructose to fructose-1-phosphate (F1P)—a step unique to fructolysis. KHKc is not regulated by ATP or other energy signals and is 10 times more active in phosphorylating fructose (Km 0.5 mM) than glucokinase (GK) is for glucose (Km 5–10 mM). Thus, most portal-circulated fructose is quickly metabolized by the liver due to KHKc’s high affinity and activity, leaving little to enter systemic circulation [19,20,22,23].

Next, F1P is split into two three-carbon molecules, dihydroxyacetone phosphate (DHAP) and glyceraldehyde, by aldolase B. Glyceraldehyde is then phosphorylated by triokinase (the third specific enzyme in fructolysis) into glyceraldehyde 3-phosphate (GAP). Both DHAP and GAP can either be used to make lactate or acetyl-CoA for new fat production, but the majority is redirected through gluconeogenesis to reform glucose [5,20,22,23,43,44,45,46,47,48,49,50,51,52,53,54]. A third important pathway will be visited in Section 2.1.3.

From an evolutionary standpoint, the KHKc mechanism (high capacity, unregulated) has been advantageous for efficiently assimilating fructose—a historically rare and sporadic component of human diets [51,54,55,56]. However, in contemporary contexts, this adaptation can present significant risks as we shall discuss further below [57,58,59,60].

#### 2.1.2. The Uric Acid Link

Figure 1 (upper left) depicts (green background) one of the consequences of unrestricted action of KHKc. In hepatocytes, fructose-1-phosphate can quickly accumulate to millimolar concentrations. Consequently, when dietary fructose intake is rapid and excessive (as with sugar-sweetened beverages and fruit juices and more so on an empty stomach), fructose phosphorylation leads to a reduction in free phosphate and increase in AMP, which in turn activates AMP deaminase and stimulates uric acid synthesis, as depicted in the figure.

Elevated intracellular uric acid levels, frequently resulting from increased fructose intake or purine metabolism, negatively impact mitochondrial structure and function through several distinct mechanisms:

Oxidative Stress Generation: Uric acid within cells promotes the formation of mitochondrial reactive oxygen species (ROS), leading to excessive oxidative stress that damages mitochondrial DNA (mtDNA) and essential proteins.

AMPK Inhibition: Elevated uric acid suppresses AMP-activated protein kinase (AMPK), a key regulator of cellular energy balance. This inhibition impairs the removal of damaged cellular components and hinders mitochondrial biogenesis.

Mitochondrial Calcium Overload: Increased uric acid activates the mitochondrial sodium-calcium exchanger, contributing to calcium overload within mitochondria.

Enzyme Downregulation: Uric acid reduces both the expression and activity of crucial enzymes such as aconitase-2 (ACO-2) in the Krebs cycle and enoyl-CoA hydratase-1 (ECoAH-1), which results in citrate accumulation. Citrate is the main transporter of carbons in the form of acetate, as substrate for DNL in the cytosol [52,53,54,55,61,62].

Excessive fructose consumption leads to hepatocyte phosphate depletion, spikes uric acid levels, fuels hypertension, and damages mitochondria. Citrate, produced in this process, further drives fat synthesis, compounding the metabolic risk.

#### 2.1.3. The Methylglyoxal Link

Excess trioses not only feed TG production (see below) but also produce toxic metabolites as well.

Figure 1 (lower left) illustrates a pathway in which fructose metabolism increases methylglyoxal (MG), a precursor of advanced glycation end products [63,64,65,66,67]. MG can modify Arg or Lys residues in proteins [68]. When fructose breakdown is not tightly regulated, excess trioses may accumulate, leading to increased liver MG production because of spontaneous DHAP decomposition. Higher DHAP also produces more G3P, which may enhance triglyceride and diacylglycerol (DAG) synthesis, and has been associated with steatosis and insulin resistance. MG generation tends to rise with elevated triose flux that supports TG synthesis [68,69,70,71]. Elevated DHAP and GA3P levels are linked to MG spikes, protein modifications, and activation of the unfolded protein response (UPR), especially when Glo1 activity is reduced. MG-modified misfolded proteins may trigger UPR responses [69,70]. For example, inositol-requiring enzyme 1 α (IRE1α) activation is associated with insulin resistance, steatosis, and inflammation through thioredoxin-interacting protein (TXNIP) and the immune response NOD-LRR and pyrin domain-containing protein 3 (NLRP3). Protein-kinase RNA-like endoplasmic reticulum kinase (PERK) may increase VLDL receptor and steatosis, while activating transcription factor 6 (ATF6) has been reported to potentially counteract lipogenesis and promote fatty acid oxidation [70,72,73].

The potential impact of excessive triose flux from fructose intake on MG production warrants further attention.

As shown in Figure 1, the detoxification of MG via the combined actions of glyoxalase I and II involves the consumption of reduced glutathione and results in the production of D-lactate. Elevated fructose consumption leads to increased concentrations of MG and D-lactate not only at the cellular level but also in serum [74,75,76]. D-lactate (distinct from L-lactate produced during glycolysis) thus serves as a circulating marker for MG synthesis and fluxes, provided input from bacteria in enteral infections can be ruled out. In this context, elevated levels have been observed in obese adults by other researchers and in adolescents by our research team [68,74,75,76,77]. Furthermore, our findings demonstrated that limiting fructose intake resulted in a 38% reduction of D-lactate within 10 days [75,76].

#### 2.1.4. DNL Converts Surplus Dietary Carbohydrates into Fatty Acids

Hepatic DNL is the metabolic pathway responsible for synthesizing new fatty acids from precursor molecules, which in turn influences both hepatic and circulating TG concentrations [35,78,79,80]. As outlined earlier, concerns regarding the metabolic effects of fructose first arose from observations of its detrimental influence on lipid homeostasis. High levels of fructose exposure can rapidly induce fasting hypertriglyceridemia, hepatic steatosis, and elevated postprandial lipemia, sometimes within only a few days [20,21,41,81,82,83].

As shown in Figure 1 (lower right), fatty acids are synthesized from acetyl-CoA, which in this case is generated via carbohydrate catabolism (through glycolysis or fructolysis) or from acetate produced by microbial fermentation of fructose. The DNL process requires specific enzymes, predominantly expressed in white adipocytes and hepatocytes [79]. It also necessitates large supplies of reduced NADPH mainly produced by the hexose-monophosphate shunt, which is fed by glucose-6-P and indirectly by concomitant fructose metabolism, as we shall detail later.

Key enzymes facilitating glucose- or fructose-driven DNL include ATP-citrate lyase (ACLY), acetyl-CoA carboxylase (ACC), and fatty acid synthase (FASN). Citrate, originating from acetyl-CoA in the mitochondrial Krebs cycle, serves as a carbon carrier to the cytosol, where ACLY cleaves it into acetyl-CoA and oxaloacetate. This cytosolic acetyl-CoA, carboxylated to malonyl-CoA, acts as the three-carbon precursor for lipogenesis. Furthermore, as indicated [78,79] previously, uric acid plays an additional role in feeding citrate to the cytosol where DNL occurs.

Sequentially, ACC—especially the cytosolic isoform ACC1—converts acetyl-CoA into malonyl-CoA. FASN then catalyzes the multi-step assembly of malonyl-CoA units into palmitate, a 16-carbon fatty acid that serves as the initial product of lipogenesis [79]. Elongases and desaturases further modify palmitate to yield various lipid species. Notably, malonyl-CoA restricts fatty acid entry into mitochondria, thereby favoring lipid synthesis over oxidation. Maintaining systemic insulin sensitivity necessitates proper balance between hepatocyte and adipocyte DNL. The expression of key lipogenic enzymes is regulated by master transcription factors such as the subject of this review, ChREBP, activated by carbohydrate intake, and sterol regulatory element-binding protein-1 (SREBP-1), induced by carbohydrate ingestion and insulin signaling [84].

DNL provides fatty acids essential for maintaining cellular structural integrity, enables storage of carbohydrate-derived energy beyond the glycogen reservoir (thereby supporting glucose homeostasis), and regulates fatty acid oxidation. Hepatic fatty acid synthesis plays a significant role in the pathogenesis of dyslipidemia and MASLD, as pointed out earlier [78,79,80,83,85,86,87]. Research investigating the metabolic consequences of increased carbohydrate, sugar, or fructose consumption demonstrates that carbohydrates—particularly simple sugars consumed in liquid form—stimulate hepatic lipogenesis [83,88]. Comparative studies further indicate that fructose is a more potent inducer of hepatic lipogenesis than glucose [52,82,89,90,91,92,93,94]. Evidence from both increased and reduced sugar or fructose intake intervention trials indicates that sugar and fructose consumption modulate hepatic DNL [83,92,95,96]. It is important to recognize that while DNL contributes newly synthesized fatty acids, the liver also incorporates fatty acids from plasma, remnant particles, and storage droplets in triglyceride synthesis. Fructose contributes to this process both by directly providing carbons for DNL and, more significantly, by amplifying the effects of glucose through the ChREBP pathway. Additionally, fructose supplies trioses that facilitate the esterification of fatty acids produced during DNL, resulting in triglyceride formation; this topic will be discussed in greater detail subsequently [17,19,20,97]. It is important to recognize that the relative contributions of fructose as a stimulus versus substrate to final triglyceride formation differ depending on specific metabolic conditions and are a matter of some controversy.

Human studies show that excessive fructose consumption leads to increased TRL and VLDL production, impacting plasma triglyceride levels [52,82,89,90,91,92,93,94,95,98,99,100,101,102,103]. Trials demonstrate that moderate-to-high fructose intake worsens metabolic outcomes, including increases in cardiovascular risk factors, visceral fat, DNL, dyslipidemia, and insulin resistance—effects not observed with glucose [42,52,82,89,90,91,92,93,94,95,98,99,100,101,102,103,104,105]. Restriction studies, especially in youth, show clear links between sugar intake typical of Western diets and adverse metabolic health, such as hepatic insulin resistance, even when not associated with weight gain [21,41,42,49,76,83]. Most interventions targeting reduced sugar-sweetened beverage (SSB) intake result in less weight gain, lower adiposity, and improved liver and insulin markers [6,106,107,108,109,110].

A possible caveat and limitation is that current evidence is primarily based on short-term intervention studies and observational findings. More research is needed to determine the long-term effects and mechanisms of sustained fructose restriction, as well as to address potential confounding factors and variability in individual responses. For instance, recent animal research reveals notable sex differences in fructose metabolism, driven by hormonal modulation of ChREBP isoforms [111]. Women show higher FGF21 levels and acute DNL sensitivity, while men develop more chronic visceral fat and hypertension [112]. Such gender differences in response require careful investigation in humans.

#### 2.1.5. A Note on the Controversy: Just Calories or Specific Effects?

Summarizing the above, the health impact of fructose is a subject of intense debate, centered on whether its harms are unique to how the liver processes it or simply the result of consuming excess calories. One viewpoint argues that fructose causes metabolic harm independently of total calorie intake due to its distinctive metabolic pathway. Unlike glucose, nearly all ingested fructose is processed by the liver. This unique metabolism leads to several consequences: (1) Excessive fructose intake can overload the liver, stimulate de novo lipogenesis, and directly generate fat molecules. (2) Fructose metabolism depletes cellular energy stores (ATP), leading to the production of uric acid, which in turn may drive high blood pressure and gout. (3) Unlike glucose, fructose does not stimulate insulin or leptin—hormones that signal satiety—potentially resulting in increased food intake [21,42,83,91,94,95,101].

An alternative viewpoint maintains that fructose is only harmful when it contributes to a caloric surplus and that it is not inherently toxic when consumed in moderate amounts. Key arguments from this perspective are that some controlled clinical trials indicate that substituting fructose for other carbohydrates does not lead to weight gain or significant metabolic issues when total calorie intake is kept constant [15,16,113,114].

In real-world diets, people rarely consume pure fructose. It is almost always ingested alongside glucose (as in sucrose or high-fructose corn syrup), which modifies its metabolic effects. When fructose is consumed in whole fruits, it comes with fiber, water, and micronutrients. These components slow absorption and make fructose not only harmless but potentially beneficial for health [8,107,108,115].

The reality lies at the intersection of dose, delivery, and overall energy balance. Fructose is not inherently toxic when consumed in moderation, nor is it exactly the same as other carbohydrates despite equal caloric content. However, when consumed in the form of sugar-sweetened beverages and ultra-processed foods, it is particularly easy to overconsume. These foods deliver a rapid influx of calories and a large fructose load to the liver at once. Consequently, fructose functions both as a driver of excess calorie intake and as a specific metabolic stressor when total calorie consumption is exceeded.

As a corollary to these considerations, the AHA advises limiting added sugars, including fructose, to no more than 6% of daily calories—a guideline that could dramatically improve metabolic outcomes and lower disease risk. Unfortunately, the reality is stark: American adolescents consume sugar at levels two-fold than recommended, with a staggering over 25% of their caloric intake coming from sugar, which is over 12.5% from fructose [8,107,108,115].

#### 2.1.6. Endogenous Fructose Production

Another source of fructose, previously believed to be restricted to certain organs such as the prostate, endogenous fructose production has now been identified in the liver, kidney, brain, and heart. Excess glucose may be converted (in part) to sorbitol (in the polyol pathway) via aldose reductase, followed by transformation into fructose through sorbitol dehydrogenase [51,53,54,56,57,109].

The quantitative role of endogenous fructose in these conditions and obesity is currently under scrutiny.

## 3. Carbohydrate Response Element-Binding Protein, ChREBP

ChREBP is a transcription factor that plays essential tissue-specific roles in fructose metabolism by regulating critical enzymes [110,111,112,113,114,116]. Within the intestine, ChREBP maintains homeostatic fructose processing, thereby preventing excessive fructose absorption into hepatocytes. In the liver, ChREBP facilitates fructose-induced lipid accumulation and toxicity. Additionally, through modulation of SREBP2 stability, ChREBP governs hepatic cholesterol biosynthesis and hepatocyte apoptosis following fructose intake [110,111,112].

### 3.1. Structural Features, Regulation

**Figure 2:**
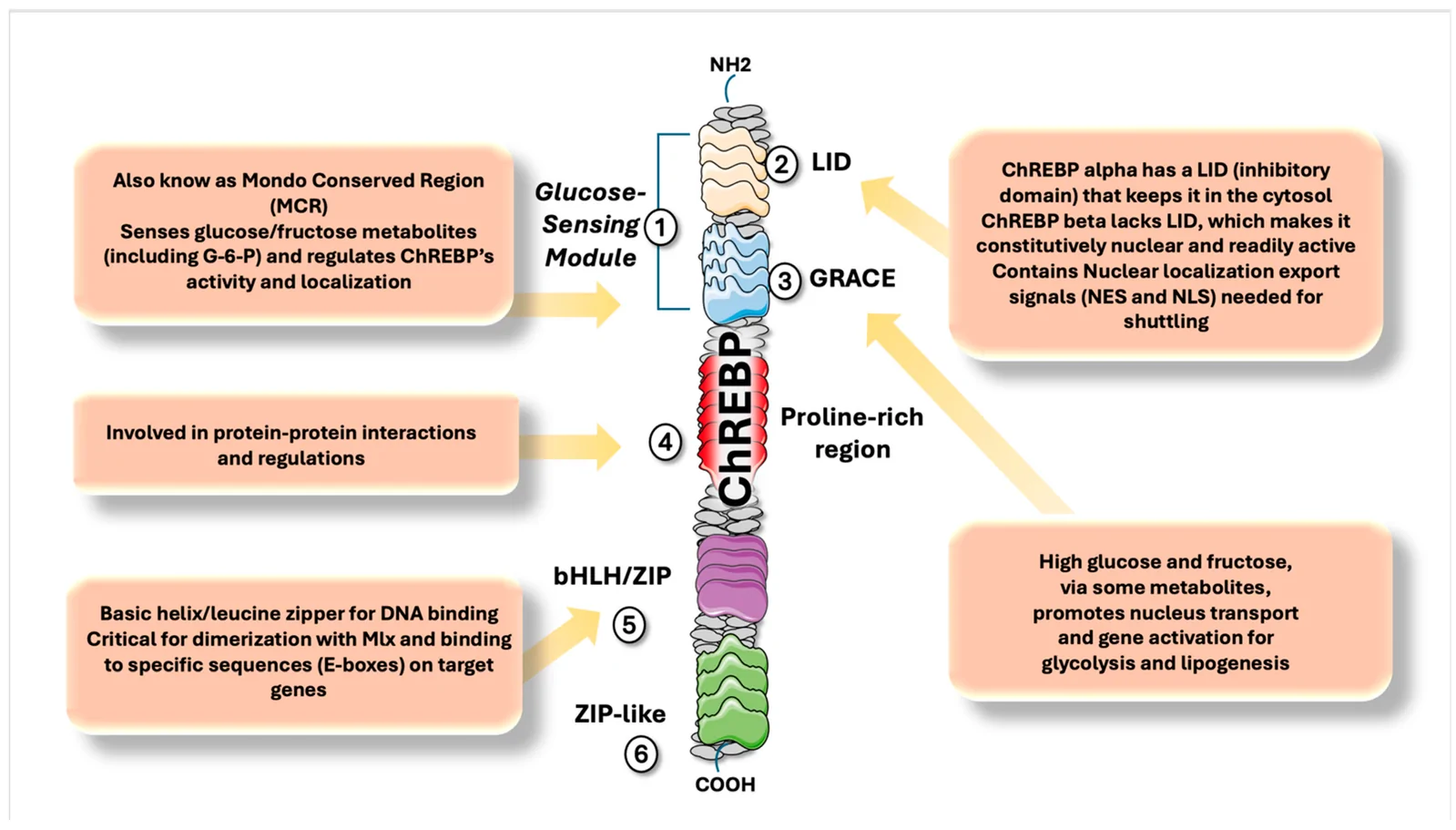
Carbohydrate response element-binding protein (ChREBP). Overview of main structural features. The N-terminal domain mainly controls where ChREBP is located within the cell and its activation in response to glucose levels. (1) Glucose-sensing module (GSM) includes two main parts: (2) the low-glucose inhibitory domain (LID) and (3) the glucose-response activation conserved element (GRACE). ChREBP exists as two isoforms: ChREBPα, which is glucose-regulated and contains all regulatory domains, and ChREBPβ, which lacks the LID. (4) Proline-rich region. The C-terminal domains feature a basic (5) helix–loop–helix–leucine zipper (bHLH/LZ) motif and (6) a dimerization and cytoplasmic localization domain. Main functions of these regions are indicated in the figure. The figures were partly generated using Servier Medical Art, provided by Servier, licensed under a Creative Commons Attribution 3.0 unported license.

From a structural point of view, ChREBP and MondoA (discovered by Uyeda’s group over two decades ago) are multi-domain proteins made up of 852 and 919 amino acids, respectively [110,116]. They have highly similar N- and C-terminal regions, which we represent in a schematic way in Figure 2. ChREBPα contains five Mondo conserved regions (MCR I–V), separated by a central proline-rich segment. The C-terminal domains feature a basic helix–loop–helix–leucine zipper (bHLH/LZ) motif and a dimerization and cytoplasmic localization domain, both of which enable them to form heterodimers with Max-like protein X (MLX) and bind DNA. MLX is a transcription factor with a critical role in metabolic regulation. The activity of ChREBP as a transcription factor depends on creating a complex with MLX that will migrate from the cytosol to the nucleus to bind to carbohydrate response elements (CHORE) in DNA and activate a menu of enzymes we shall describe later [24,117,118,119,120].

The N-terminal domain mainly controls where these proteins are located within the cell and their activation in response to glucose levels. A key regulatory region here is the glucose-sensing module (GSM), which functions similarly in both proteins. As depicted in the Figure, this module includes two main parts: the low-glucose inhibitory domain (LID) and the glucose-response activation conserved element (GRACE), which span MCR I–IV and MCR V, respectively.

• The GRACE domain drives transactivation, while the LID blocks this function when glucose is low [26,121].
• When glucose rises, the inhibition by LID is lifted, allowing transcriptional activation. ChREBP exists as two isoforms: ChREBPα, which is glucose-regulated and contains all regulatory domains, and ChREBPβ, which lacks the LID domain and remains transcriptionally active regardless of glucose levels (see Table 1).
• Deleting any single part of MCR I–IV eliminates the proteins’ ability to activate transcription in response to glucose, indicating that all four regions are needed. The specific spacing between MCR II, III, and IV supports the structure of this functional area [24,117,118,119,122,123,124,125].
• Repression of GRACE by LID likely involves an internal interaction between MCR I–IV and MCR V, causing a shape change that stops DNA binding and activation. If this interaction is disrupted, glucose metabolites can directly bind to the MCR I–IV region.
• In addition, G6P binds allosterically in the highly conserved MCR VI region, located within the GRACE domain.

Structural studies of ChREBP have identified crucial regulatory elements: two CRM1-dependent nuclear export signals (NES1, NES2), a bipartite nuclear localization signal (NLS) that mediates nuclear import, and constitutive binding to 14–3–3 proteins for cytoplasmic retention. Both MondoA and ChREBP are pivotal in regulating metabolic homeostasis, especially glucose and lipid metabolism, but their dysregulation under nutrient excess can lead to obesity and diabetes.

• ChREBPβ remains active regardless of glucose levels, is constitutively nuclear, and is much more potent. Its expression rises as carbohydrate metabolism activates ChREBPα, creating a feedforward loop [28,30,37,69,114,126,127].
• ChREBPβ is produced from an alternate promoter, lacks the LID domain and nuclear export signals, and is primarily nuclear with higher transcriptional activity than ChREBPα.
• When cellular glucose metabolites rise, they activate ChREBPα, initiating a feedforward loop that increases ChREBPβ expression; ChREBPβ can also enhance its own production via its promoter’s ChoREs.
• High glucose or fructose intake elevates hepatic ChREBPβ in both mice and humans, with fructose causing greater activation than glucose. We shall delve into the precise known mechanisms in Section 4 [117,128,129,130,131,132].

### 3.2. ChREBP Initiates Multiple Biological Pathways

**Figure 3:**
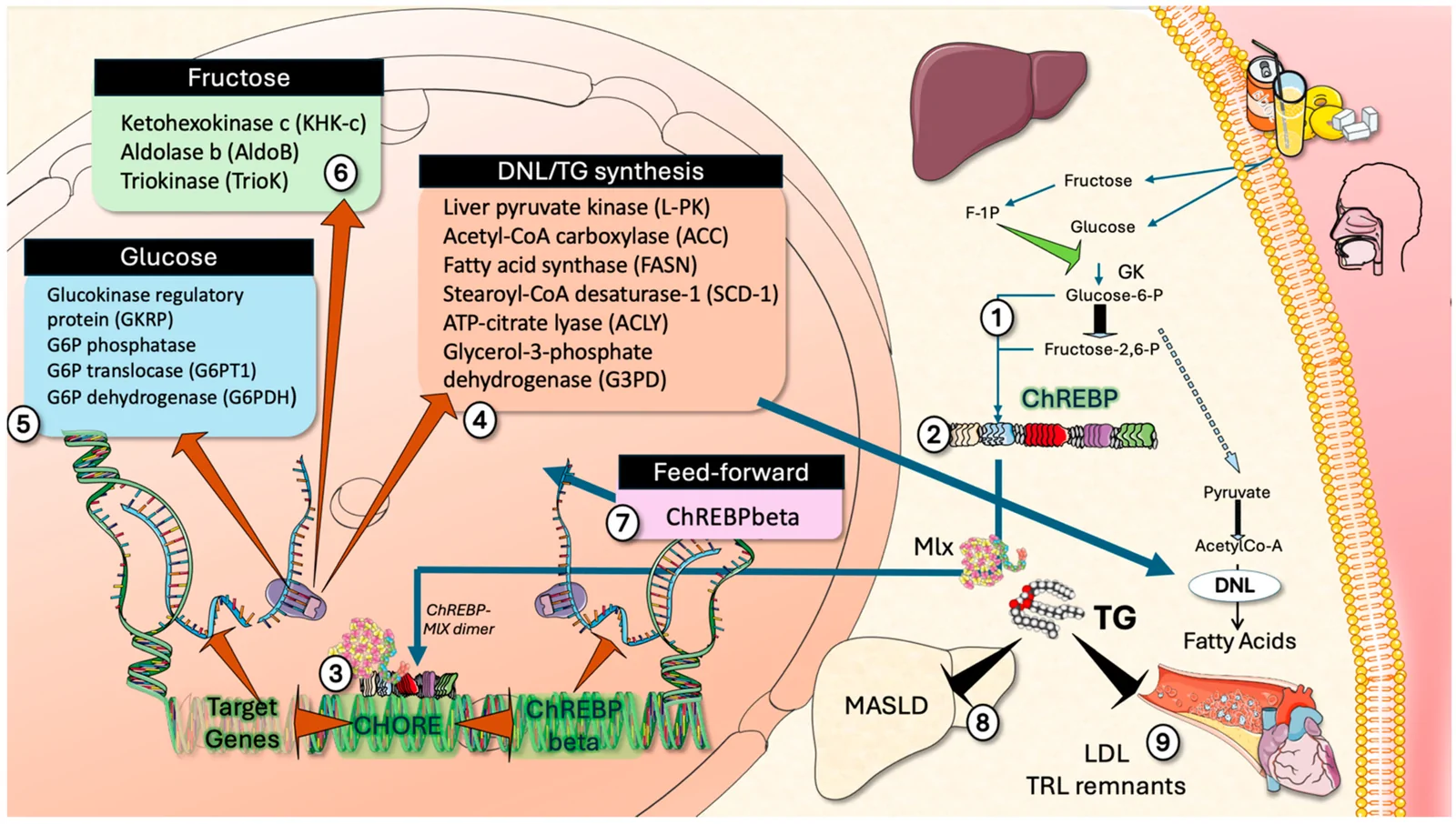
Hepatic ChREBPα, activation by sugar, and its main physiological actions. (1) Upon sugar loads, glucose-6-P and fructose 2,6-P activate (2) GRACE (3), leading to dimerization with Mlx and binding to carbohydrate response element (CHORE) in DNA. Consequently, different sets of enzymes (as shown) are expressed: (4) DNL and TG synthesis; (5) glucose metabolism; (6) fructose metabolism; and (7) feedforward ChREBPβ. A key end result is TG production potentially leading to (8) metabolic-associated steatotic liver disease (MASLD) and (9) triglyceride-rich lipoproteins (TRL) dyslipidemia. The figures were partly generated using Servier Medical Art, provided by Servier, licensed under a Creative Commons Attribution 3.0 unported license.

As depicted in Figure 3, ChREBP binds Mlx, and the resulting dimer activates gene expression upon nuclear translocation [119]. It targets genes involved in four main pathways: (1) de novo lipogenesis, such as ACC, FASN, and SCD1; (2) glucose; (3) fructose; and (4) feedforward mechanisms by activation of ChREBPβ transcription.

**Figure 4:**
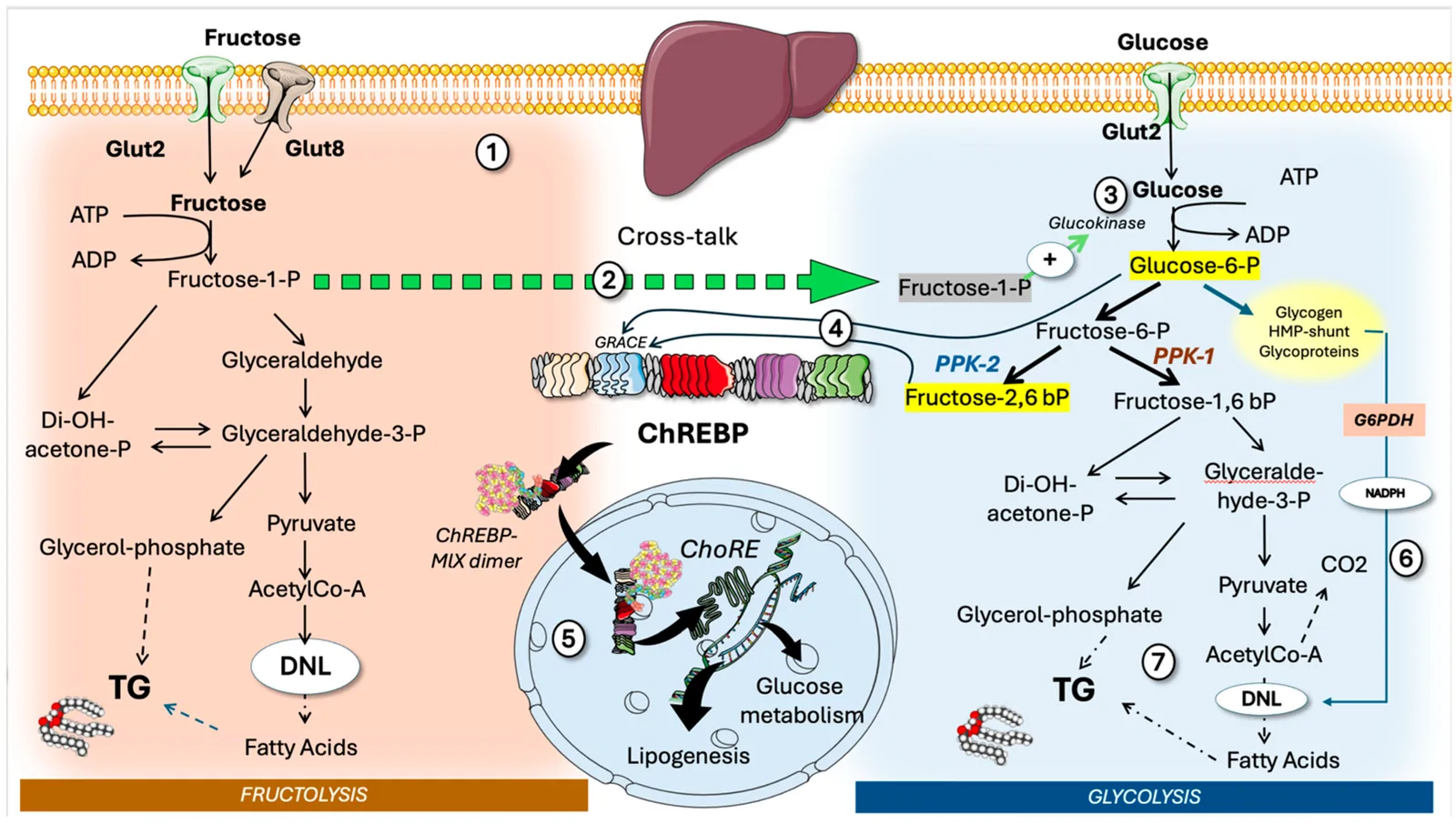
Crosstalk of fructose and glucose metabolism in the liver: potentiation of ChREBPα activation. Fructose metabolism (1), as previously shown, has a first phosphorylation step leading to (2) fructose-1-P. Concomitant glycolysis is initiated by glucokinase (3). Fructose-1-P dissociates glucokinase kept in the nucleus, inhibited by its binding to glucokinase regulatory protein (GKRP, see more detail in Figure 5). Glycolysis is stimulated, and its metabolites (4) glucose-6-P and fructose 2,6bP, acting on GRACE, enhance (5) ChREBP dimerization with Mlx and translocation to the nucleus, where it activates metabolism, as shown in Figure 3. Glucose-6-P provides (6) NADPH through the action of glucose-6-P dehydrogenase (G6PDH) for DNL. The figures were partly generated using Servier Medical Art, provided by Servier, licensed under a Creative Commons Attribution 3.0 unported license.

Increased hepatic glucose metabolism promotes lipogenesis via ChREBP activation through glucose metabolite binding, as explained in detail in Section 4. As shown in Figure 4, ChREBP triggers the expression of DNL pathway genes [117,118,119,120,125,128]. Deleting hepatic ChREBP in mice reduces steatosis but leads to insulin resistance and impaired glucose tolerance, possibly due to decreased expression of glucokinase regulatory protein (GKRP), glucose-6-phosphatase (G6PC, key enzyme for hepatic glucose export), together with glucose-6-phosphate transporter 1 (G6PT1), which mediate glucose export from hepatocytes [117,118,119,120,125,128]. Overexpression of ChREBP increases both lipogenic gene expression and hepatic steatosis. In MASLD patients, ChREBP expression is higher in those with significant steatosis, suggesting its central role in glycolytic overload-induced hepatic fat accumulation [26].

### 3.3. ChREBP Activation Also Contributes to the Development of Hepatic Insulin Resistance

In healthy humans, insulin receptor substrate 2 (IRS-2)/Akt2 signaling suppresses hepatic glucose output and promotes glycogen/lipid storage. In MASLD, selective insulin resistance impairs IRS-2/glucose-suppressive pathways but not IRS-1/lipogenic ones. Increased glucose and fructose metabolism, activating ChREBP, downregulate IRS-2, which—along with hyperinsulinemia—promotes hepatic insulin resistance and MASLD [127].

Indeed, in obese ob/ob mice, reduced IRS-2 and increased G6PC mRNA were observed; hepatic ChREBP knockdown normalized these changes, suggesting ChREBP-mediated regulation of IRS-2 may be involved in insulin resistance and MASLD development [127].

### 3.4. Additional Allosteric Activators of ChREBP

Beyond the direct role of fructose via ChREBP, metabolites released by the gut upon substantial fructose exposure can provide substrates for hepatic DNL involving ChREBP. Short-chain fatty acids (SCFAs) and amino acids produced by gut microbiota from unabsorbed dietary fructose act locally to regulate intestinal homeostasis or are transported to the liver via the portal vein as lipogenic substrates [25,118,120,125]. Acetate, a gut-derived SCFA generated during excessive fructose consumption, supports hepatic DNL through its conversion to acetyl-CoA by acyl-CoA synthetase short-chain family member 2 (ACSS2), an enzyme regulated transcriptionally by ChREBP [25,118,120,125]. In this context, ChREBP detects fructose entering hepatocytes and modulates key enzymes responsible for transforming gut-derived acetate into acetyl-CoA, a lipogenic substrate [25,31,133]. Given ChREBP’s involvement in both intestine and liver, its homeostatic regulation in the intestine ensures efficient fructose metabolism under normal intake, whereas excessive fructose consumption robustly stimulates hepatic ChREBP, orchestrating metabolic responses from both hepatic and extrahepatic sources to exacerbate MASLD and associated toxicity.

In summary, ChREBP is recognized as the main glucose-sensing regulator driving hepatic de novo lipogenesis, acting as a central point for lipid synthesis in the liver, driven by concomitant fructose metabolism. Disrupting this lipogenic feedforward loop by modifying ChREBP or through other avenues described in Section 5 is viewed as a promising strategy to develop new treatments for MASLD. As outlined in Section 4, the interactions and potentiation resulting from enhanced hepatic exposure to fructose warrant careful examination to better understand ChREBP biology and identify potential therapeutic approaches. A key point to be mentioned is that ChREBP-β has distinct tissue-specific roles: intestinal ChREBP-β facilitates fructose absorption, preventing intolerance and gut issues; hepatic ChREBP-β activates when intestinal capacity is exceeded, driving lipogenesis and metabolic adaptation to limit oxidative stress and energy deficits [24,125,126,127]. This duality presents a key translational limitation. Indeed, inhibition of ChREBP may produce severe gastrointestinal and systemic side effects. Therefore, therapeutic strategies should prioritize strict liver-specific targeting to avoid disrupting ChREBP’s essential roles elsewhere.

## 4. The Hepatic Fructose–Glucose–ChREBP Pathway and an Emerging New Mechanism

### 4.1. Comparative Analysis of Parallel Glucose and Fructose Metabolic Pathways: Similarities and Differences

The effects of fructose on hepatic metabolism should be assessed considering that both fructose and glucose generally enter the liver simultaneously and in similar amounts, regardless of whether their source is sucrose or HFCS. This combination creates an optimal environment for metabolic disruption, particularly with respect to increased lipid synthesis and the development of insulin resistance. Thus, as seen in Figure 4, where we compare both glucose and fructose main metabolic pathways, the stronger effect of fructose compared to glucose in activating ChREBP is likely due to the difference in the pathways and enzymes involved in processing each sugar within the liver. As outlined in Section 2, the liver contains KHKc, which [134,135,136,137,138] allows the liver to extract most of the ingested fructose during its first pass [134,135,136,137,138]. Fructose-derived substrates enter the triose phosphate pool, where they may undergo conversion to glucose-6-phosphate (G6P) via gluconeogenesis or be further metabolized to pyruvate through glycolytic pathways [134,135,136,137,138]. In contrast, hepatic glucose phosphorylation following meals is regulated by glucokinase, which typically remains inactive in the nucleus due to its binding with the glucokinase (GK) regulatory protein (GKRP). GK is a high-Km, high-output enzyme [34,69,116,127,139]. At low-glucose input, glucose is metabolized by hexokinase, a very-low Km, quickly saturated enzyme. As a result, only a limited proportion of ingested glucose undergoes initial hepatic processing, potentially minimizing activation of hepatic ChREBP when glucose is consumed independently. Although oral ingestion of glucose alone does not significantly stimulate hepatic ChREBP, such activation can occur if glucokinase is induced.

Precisely, a very potent modulator of GK, promoting its detachment from GKRP, is the first fructose metabolite, F1P, as depicted in the figure.

### 4.2. Crosstalk Between Fructose and Glucose Metabolism in the Liver: The Role of F1P as a Signaling Molecule Linked to Modern Dietary Habits

As illustrated in Figure 4, fructose needs only one, unregulated, high-capacity phosphorylation step to produce fructose-1-phosphate. F1P acts as a signaling molecule, activating glucose metabolism by influencing GK and pyruvate kinase as well [140]. These activations increase metabolic intermediates, which, after glycogenesis is saturated, ultimately drive DNL and TG synthesis. Consequently, fructose-1-phosphate facilitates DNL via the mass action law, influencing not only fructose but also glucose metabolic pathways [20,23,87,93,141]. As previously outlined, this mechanism is accompanied by heightened activation of ChREBPα, which subsequently drives the expression of DNL enzymes, glycolytic and fructolytic enzymes, along with feedforward processes such as ChREBPβ expression, as illustrated in Figure 3.

### 4.3. Allosteric Modulators of GRACE in ChREBP

Multiple glucose metabolites have been proposed as the allosteric modulators of GRACE in ChREBP. Glucose-6-P and fructose-2,6 bisP appear so far as the most important. As shown in Figure 4, fructose-2,6 bisP is the product of F-6-P phosphorylation by phospho-fructokinase 2 (PPK-2) instead of the canonical PPK1 of glycolysis that produces fructose 1,6 bisP. Fructose-2,6 bisP also serves as a classical allosteric modulator of glycolysis-gluconeogenesis [34,134,139,142,143,144].

In summary, glucose metabolism that relies on GK in liver cells (which in turn is made readily available in the cytosol when F1P is present) makes them prone to unchecked glycolysis and metabolic overload. Increased levels of G6P and F26BP activate ChREBP/Mlx, which leads to the upregulation of genes associated with glycolysis, gluconeogenesis, and lipogenesis—ultimately promoting hepatic steatosis and insulin resistance. In patients with MASLD, enhanced expression of lipogenic genes coincides with elevated GK and ChREBPβ. The activation of ChREBP supports fatty acid production for energy storage and heightens G3PD expression, crucial for providing the backbone of triglycerides. Furthermore, it stimulates GKRP, G6PC, and G6P translocase, aiding in the regulation of G6P concentrations and facilitating glucose release from the liver [134,139,142,143,144].

### 4.4. ChREBP and Insulin Resistance

**Figure 5:**
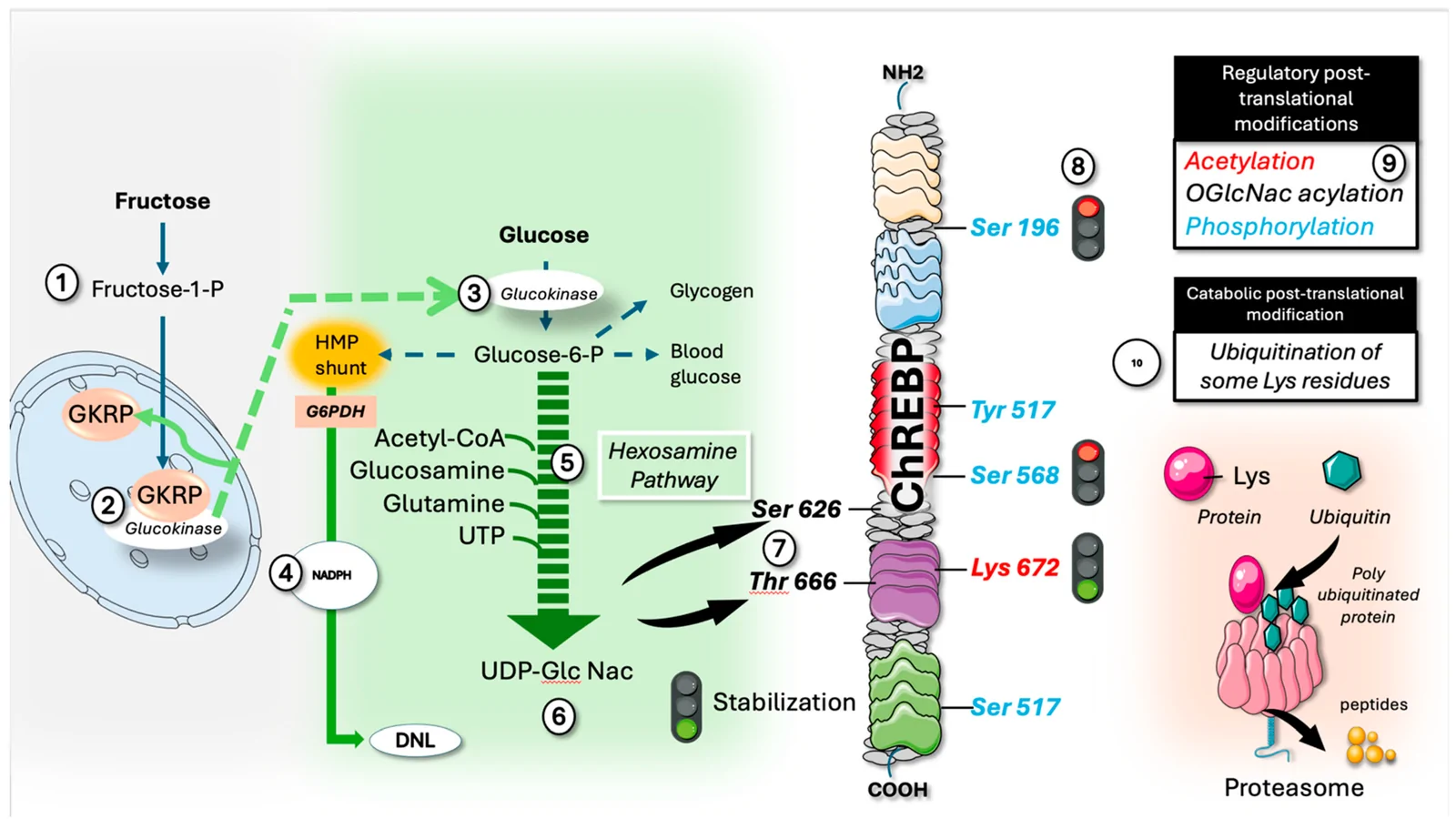
ChREBPα is also regulated by posttranslational modifications. Role of fructose metabolism. (1) Fructose-1-P dissociates glucokinase (GK) kept in the nucleus, inhibited by its binding to (2) glucokinase regulatory protein. GK (3) produces glucose-6-P, which is the main metabolic crossroads that not only provides (4) NADPH for DNL but feeds the (5) hexosamine pathway that produces (6) UDP-Glc Nac (UDP-*N*-acetyl glucosamine), which is needed for (7) OGlc Nac acetylation of serine or threonine residues, leading to stabilization of ChREBPα. Other posttranslational modifications (9) are acetylation (in red) and phosphorylation (blue); the latter inhibits ChREBPα during fasting. Ubiquitination (10) acting on lysine residues plays a role in proteolysis and shortening ChREBPα half-life. The figures were partly generated using Servier Medical Art, provided by Servier, licensed under a Creative Commons Attribution 3.0 unported license.

In turn, excessive hepatic glucose and fructose metabolism promotes insulin resistance via glycolytic overload, including hexosamine pathway activation (see Section 5), stabilization of GK and ChREBP (which suppresses insulin receptor substrate 2 (IRS-2)), and increased MG triggering the UPR, as shown in Figure 1 [36,105,145]. As shown in Figure 4 (and further developed in Figure 5), glucose-6-P is a key crossroads between glycolysis, glucose output, glycogen synthesis, the hexosamine pathway, and the hexose-monophosphate shunt. The latter provides cytosolic-reducing equivalents as NADPH, which are indispensable to reduce acetyl-CoA carbons for FA synthesis. Additionally, uric acid produced from rapid fructose metabolism is thought to promote DNL, as depicted in Figure 1. Even small amounts of fructose significantly elevate hepatic glucose. To complete the picture, research also suggests that fructose-1-P inhibits glycogen phosphorylase and activates pyruvate kinase, the last step of glycolysis [25,36,69,112,127]. While fructose was once thought to be more lipogenic than glucose due to distinct metabolic pathways, additional mechanisms, such as relief of GKRP inhibition by F1P, may contribute to glycolytic overload and steatosis [25,140,146].

Thus, the primary mechanism behind fructose-driven DNL in the liver is the rise in hepatic fructose-1-P, which causes dissociation of GK from its inhibitory partner. GKPR fructose-1-P likely evolved as a beneficial signaling molecule for efficiently processing a rare dietary sugar, but this adaptation is now problematic given today’s abundant fructose intake [140].

### 4.5. ChREBP and SREBP1c

ChREBP works alongside sterol regulatory element-binding protein 1c (SREBP1c), which also responds to nutrients and hormones to enhance lipogenesis [28,80,118,120,121,131,141,147,148]. Insulin significantly increases SREBP1c activity through AKT and mTORC1 signaling. Chronic fructose consumption may elevate SREBP1c activity due to increased adiposity and hyperinsulinemia. Other metabolic transcription factors, including peroxisome proliferator-activated receptor (PPAR) alpha or gamma, estrogen-related receptors, and liver X receptor, are coactivated by peroxisome proliferator-activated receptor gamma coactivator 1b (PGC-1b), which supports multiple sugar-induced metabolic changes [28,131,141,147,148].

### 4.6. Interplay KHK-C/ChREBP

KHK-c and ChREBP form a two-way regulatory loop essential for fructose and lipid metabolism. ChREBP boosts KHK transcription directly by binding to carbohydrate response elements in the KHK gene promoter, affecting both intestinal and hepatic expression. Loss of ChREBP lowers KHK and Glut5 levels, leading to fructose intolerance. As previously mentioned, KHK-driven fructose metabolism produces uric acid, which promotes ChREBP nuclear translocation and increases KHK transcription. Additionally, KHK activity triggers signals needed for ChREBP activation from its inactive cytosolic state.

### 4.7. ChREBP Is Also Regulated by Posttranslational Modifications

Beyond the transcriptional activation and allosteric modulation of ChREBP described so far, a lot of attention is paid as well to posttranslational modifications of this transcription factor, as depicted in Figure 5 (right side). Allosteric activation of ChREBP is necessary for its carbohydrate-sensing role, but posttranslational modifications can affect its stability, localization, and interactions, altering transcription efficiency.

For instance, in hypoglycemia, phosphorylation reduces ChREBP’s nuclear entry and DNA binding, while abundant carbohydrates cause acetylation and O-GlcNAcylation, increasing binding, stability, and activity [149,150]. Lipid droplets and associated proteins may also sequester ChREBP, limiting its activation. More regulatory mechanisms are likely to emerge.

Fructose, as previously indicated, stimulates glycolysis through the action of F1P and promotes the release of GK from GKRP. This process channels G-6-P into critical metabolic pathways, one of which is the hexosamine monophosphate pathway (Figure 5). This pathway plays a pivotal role in nucleotide, amino acid, lipid, and sugar metabolism. It generates UDP-GlcNAc, which is utilized for *N*-acetylation of the serine and tyrosine hydroxyl groups in proteins, conferring various regulatory functions [38,122]. In relation to ChREBP, this modification stabilizes and enhances its activity during nutrient intake. The resulting effect is synergistic and may lead to a significant increase in ChREBP activity driven by the other allosteric mechanisms described.

A similar effect is known to occur by acetylation of Lys 672. Conversely, phosphorylation by AMPK and other kinases during low CHO reduces the stability, thus curbing CHREBP activity [28,118,147].

### 4.8. Ubiquitination

**Figure 6:**
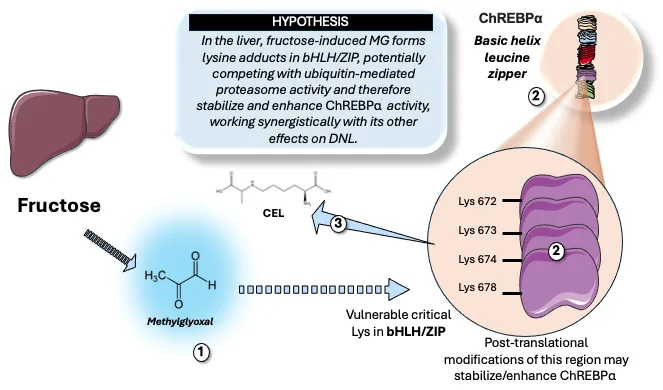
Hypothetical alternate mechanism for ChREBPα stabilization and increased activity. Fructose metabolism (as shown in Figure 1) produces methylglyoxal (MG), a very reactive dicarbonyl (1). The basic helix leucine zipper on ChREBPα (2) contains at least four vulnerable Lys residues (one of which is used as a regulating target for acetylation). We hypothesize that (3) CEL (carboxyethyl lysine) could be formed by adduct formation between MG and one or more of these available lysines, preventing ubiquitination and proteolysis. Stabilization would increase the duration of ChREBPα activity, enhancing its lipogenic effect. The figures were partly generated using Servier Medical Art, provided by Servier, licensed under a Creative Commons Attribution 3.0 unported license.

Recent studies indicate that a high-fructose diet may impair ubiquitination [151]. Ubiquitination is a posttranslational modification, wherein the small protein ubiquitin (Ub) is covalently attached to specific lysine residues on substrate proteins, playing a pivotal role in processes such as protein degradation, cellular signaling, and trafficking. This modification relies on a highly conserved enzymatic cascade involving three distinct enzymes: E1 (ubiquitin-activating enzyme), E2 (ubiquitin-conjugating enzyme), and E3 (ubiquitin ligase). The cascade culminates in the formation of an isopeptide bond between the C-terminal glycine of ubiquitin and the ε-amino group of a substrate lysine. As shown in Figure 5, poly-ubiquitinated proteins are targeted to the proteasome for hydrolysis. At the molecular level, E3 ligases align the substrate lysyl residue so the ε-amino group executes a nucleophilic attack on the thioester bond connecting ubiquitin to the E2 cysteine. This reaction deprotonates the lysine, enabling it to break the E2–Ub linkage and form a stable isopeptide bond between the ubiquitin C-terminus and the lysine residue. As shown in Figure 6, ChREBP basic helix leucine zipper region has at least four Lys residues that can potentially be targets for ubiquitination, which could modify the half-life of this transcription factor. Indeed, as described earlier, Lys 672 is a key site for acetylation, which stabilizes ChREBP [24,117,118,121].

A study identifies DNA-binding protein 1 (DDB1) as a fructose-responsive protein that associates with ChREBPα. The findings demonstrate that excessive fructose consumption enhances the stability of ChREBPα by inhibiting its ubiquitination and subsequent degradation [33,152]. A comprehensive review catalogues various PTM influencing ChREBP activity. It notes that ubiquitination commonly results in ChREBP degradation, mediated by E3 ligases such as DDB1. Specifically, the DDB1 E3 ligase orchestrates this effect through cryptochrome-1 (CRY1), a well-established ubiquitination substrate. It has been shown that a fructose diet can impair this ubiquitination, thereby augmenting ChREBP protein levels and activity [151,152].

Thus, reducing ubiquitination of ChREBP is pro-lipogenic, and these have been some of the mechanisms proposed.

### 4.9. Hypothesis: Does the Overflow of Trioses to Methylglyoxal (MG) Induced by Fructose Contribute to the Stabilization of ChREBPα?

#### 4.9.1. What Is Known: Structural Findings

As shown previously in Figure 1 and Figure 3, trioses from fructolysis and glycolysis drive both lipogenesis and MG production, leading potentially to Glo pathway overload, elevated MG levels, and possible protein damage. MG is notably reactive, exhibiting approximately 250 times greater reactivity than glucose [62,68,153,154,155,156,157,158]. Many studies emphasize its significant role in intracellular glycation, a process that has often been primarily associated with glucose, but it should be better considered as fueled by fructose, particularly in Western dietary patterns [62,68,153,154,155,156,157,158].

In vitro and animal studies show that MG alters protein targets and disrupts cellular pathways [63,66,157,158,159].

• MG modifies histones H3 and H4 (e.g., H3K4 and H3R2), consequently influencing gene expression.
• MG also modifies GAPDH, resulting in altered functionality and inhibition of Notch1 translation.
• Methylglyoxal (MG) modifies mSin3A and Hsp90, thereby influencing cancer-associated signaling pathways. It activates Hsf-1 and Nrf2, promoting cellular stress and adaptive responses.
• MG impairs albumin’s function, diminishes collagen-binding capacity, and decreases proteasome activity.
• Additionally, MG affects Notch1 mRNA translation as well as VEGF and Ang-2 expression.

Collectively, these examples demonstrate that adduct modifications by MG are associated with decreased protein degradation, compromised chaperone activity, and activation of inflammatory pathways, often involving RAGE mediation [62,63,64,65,67,68,69,73,160].

#### 4.9.2. What Is Known: Human Studies

A growing body of clinical evidence robustly demonstrates that methylglyoxal (MG) fluxes are markedly elevated in a range of metabolic disorders. Multiple independent research groups, including our own, have consistently observed significantly higher MG levels in individuals with obesity as well as in subjects consuming high-fructose diets [64,66,67,71]. Furthermore, these levels are responsive to intervention. Adult obesity is associated with elevated serum MG and D-lactate levels [68,77]. Limited research has assessed MG in the context of obesity or metabolic syndrome because of technical difficulties; however, plasma D-lactate concentrations provide an indirect measure (see Figure 1), provided intestinal sources can be ruled out, such as in intervention studies. Indeed, in our 9-day isocaloric fructose restriction study on adolescents, D-lactate was linked to baseline liver fat fraction (*p* < 0.001) and visceral adipose tissue (*p* < 0.001), but showed no association with subcutaneous adipose tissue [75]. At the start, D-lactate was positively correlated with DNL-area under the curve (AUC) (*p* = 0.003), liver fat fraction (*p* = 0.02), triglycerides (TG) (*p* = 0.004), and TG/high-density lipoprotein ratio (*p* = 0.002). Following 9 days of isocaloric fructose restriction, serum D-lactate levels dropped by 50% (*p* < 0.0001), and changes in D-lactate were tied to changes in both DNL-AUC and measures of insulin sensitivity. The association between baseline D-lactate, DNL, and insulin sensitivity measures, along with the significant drop in D-lactate after 9 days of isocaloric fructose restriction, indicates that DNL and nonenzymatic glycation are functionally connected through intermediary glycolysis in metabolic syndrome development. This highlights fructose as a key dietary factor influencing both pathways [19,20,41,70,72,93,161]. It must be noted that D-lactate assay has a limitation produced by its other main source, the intestines. D-lactate is produced in the colon by bacterial species possessing D-lactate dehydrogenase (D-LDH), such as Lactobacillus, Weissella, Bifidobacterium, Streptococcus, Escherichia coli, and Klebsiella pneumoniae, among others.

Under healthy physiological conditions, D-lactate accumulation is minimal; the majority is converted into short-chain fatty acids (SCFAs) that supply energy to colonic mucosal cells. Small quantities are absorbed via monocarboxylate transporters and are efficiently eliminated by hepatic mitochondrial metabolism.

Compromised intestinal function may lead to excessive D-lactate production, resulting in potential toxicity. In these cases, elevated systemic levels of D-lactate indicate disruption of the intestinal barrier, for example, during ischemic events, and can serve as an early biomarker for mesenteric injury.

#### 4.9.3. What Is Not Known

Given that MG has been shown to disrupt protein degradation, impair chaperone function, and activate inflammatory pathways, it logically follows that, alongside the allosteric and translational effects previously described, concurrent fluctuations in MG concentration after high fructose and glucose intake (from sugar or HFCS) suggest studies to explore its impact on ChREBPα activity.

#### 4.9.4. Hypothesis

Considering the evidence discussed above, we hypothesize that acute elevations in MG, resulting from increased hepatic fructose metabolism, may facilitate the formation of adducts with lysine residues on ChREBPα, as depicted in Figure 6. This molecular interaction may yield a rapid and potentially synergistic stabilization of ChREBPα, thereby enhancing its activity over time. Specifically, we advance the hypothesis that elevated hepatic concentrations of both fructose and MG can induce episodic lysine modifications that impair the ubiquitination machinery, ultimately stabilizing ChREBPα and delaying its proteolytic degradation. While this synergistic double-impact mechanism may offer a new framework for understanding how fructose may potentiate ChREBPα activity and its downstream metabolic consequences, it is important to emphasize that this remains a hypothesis.

#### 4.9.5. Proposed Speculative Mechanism

We posit that rising MG from fructose metabolism can form adducts with ChREBPα lysine residues, temporarily stabilizing ChREBP. Rigorous experimental and clinical studies are necessary to validate the causal relationships and delineate the precise molecular pathways involved. One potential consideration is that this Lys modification could trigger additional degradation pathways, such as chaperone-mediated autophagy, independent of ubiquitin. Nevertheless, these mechanisms typically proceed at a slower rate. Regardless, it is important to investigate this matter thoroughly.

A special case should also be considered. As shown in Figure 5, lysine 612 (Lys612) is a crucial regulatory site within the low-glucose inhibitory domain (LID) of ChREBP. Its acetylation limits ChREBP activity during low glucose, while mutation or loss of acetylation increases nuclear localization, DNA binding, and gene expression. Lys612’s modification also affects protein interactions by controlling cofactor binding. Unlike Lys672, which promotes ChREBP activation during high glucose via p300 acetylation, Lys612 acts as a restraint under low nutrient conditions. A potential added effect of MG, beyond its hypothetical stabilization of ChrEBP, could therefore be the impeachment of the acetylation of Lys612 and the release of the inhibitory effect.

#### 4.9.6. Workflow to Test the Hypothesis

To examine whether fructose-derived MG glycation stabilizes ChREBP via lysine modifications, a suggested progressive workflow is outlined below:

Determine if MGO Modifies Lysine Residues on ChREBP

• In Vitro Assays: Incubate recombinant ChREBP with varying MG concentrations. Use LC-MS/MS to identify MG-lysine adducts and Western blots with anti-CEL/MG antibodies.
• Cellular Assays: Treat hepatocytes with MG or Glyoxalase 1 inhibitor, immunoprecipitate ChREBP, and confirm modifications via antibody detection.

Assess Whether MGO Modifications Stabilize ChREBP

• Cycloheximide Chase: Measure ChREBP half-life after MG treatment using time-course Western blots.
• Ubiquitination and Proteasome Assays: Co-transfect cells with tagged constructs. MG treatment should decrease poly-ubiquitinated ChREBP. Use MG132 to test proteasomal degradation impact.
• Site-directed Mutagenesis: Mutate lysines identified. Test if stabilization effect is lost after MG exposure.

Test Whether Fructose Reduction Mitigates This Pathway

• Cellular Studies: Reduce fructose in culture. Monitor MGO levels and CEL modifications by LC-MS/MS and ChREBP IP.
• Genetic Invalidation: Knock down KHKc and observe effects on MG and ChREBP in high-fructose conditions.
• Animal Models: Feed mice high-fructose diets and then split them into continued high-fructose or low-fructose groups. Analyze hepatic MG, ChREBP modifications, and lipogenic gene expression post-intervention.

## 5. Future Developments

Given the evidence presented above, it is advisable to mitigate ChREBPα overactivation by limiting fructose intake or by targeting the fructose metabolic pathway. In addition, therapeutic approaches designed to modulate ChREBPα expression or activity are under ongoing investigation.

### 5.1. Curbing Fructose Metabolism Upstream: KHKc Inhibitors

Previously, we discussed the significance of KHKc in fructose metabolism linked to IR, obesity, type 2 diabetes, and MASLD. Therefore, inhibiting KHKc presents an attractive treatment option for these conditions. So far, three substances targeting KHK have advanced to clinical trials. Pfizer developed the most advanced clinical KHK inhibitor, PF-06835919, which proved generally safe and well tolerated across tested doses [137,162,163,164]. This agent was included in several phase 2 trials (ClinicalTrials.gov Identifiers: NCT05463575, NCT03969719, NCT03256526, and NCT06089265).

Recent Phase 2 Clinical Trial Results for PF-06835919: Hereditary Fructose Intolerance (HFI): The trial included HFI patients, showing that a daily 300 mg dose for 9 days improved fructose tolerance in multiple organs. The drug blocked toxic F1P buildup, preventing hypoglycemia and ATP loss. Liver Fat Mechanism (MASLD): Imaging confirmed suppression of fructose phosphorylation in the liver. Pfizer has stopped internal monotherapy development for MASH/NASH, now focusing on niche metabolic diseases (like HFI) and combination therapies. Eli Lilly has begun phase 1 trials for two KHK inhibitors aimed at diabetes and NASH (ClinicalTrials.gov Identifiers: NCT04559568 and NCT04270370). Another potent zwitterionic KHKc inhibitor, BI-9787, is undergoing animal testing [137,162,163,164]. Administering pharmacological KHK inhibitors blocks fructose metabolism. This blockage successfully prevents ChREBP activation, reversing fructose-induced de novo lipogenesis, hypertriglyceridemia, and hepatic steatosis.

### 5.2. Modulating Fructose Metabolism Downstream: Strategies Involving Methylglyoxal Quenchers and Glo1 Enhancers

If our hypothesis is correct—namely that MG functions to stabilize ChREBP and thereby extend its activity—then agents that quench MG or activate Glo may be considered as viable therapeutic options.

Although molecules like aminoguanidine and phenacylthiazolium bromide are potent MG scavengers, they were found to be hazardous and unstable, respectively [160]. Enhancing Glo1 expression and activity offers a more feasible and potentially effective approach. Glo1 efficiently counters MG-related dicarbonyl stress by metabolizing MG at diffusion-limited rates [165]. Small-molecule activators of the transcription factor nuclear factor-erythroid factor 2-related factor 2 (Nrf2) can stimulate Glo1 expression [63,65,66,67,127,160]. Among such compounds, trans-resveratrol gave the strongest maximal response in Glo1-ARE (antioxidant response element) transcriptional activity, while hesperetin showed the lowest median effective concentration. Combining these two substances resulted in pharmacological synergy. The first clinical trial using a Glo1 inducer, the combination of trans-resveratrol and hesperetin (tRES-HESP), was completed successfully as a randomized, double-blind, placebo-controlled crossover phase 2A study. tRES-HESP reduced low-grade inflammation [127,160].

### 5.3. Drugs Acting on ChREBP

Inhibitors of de novo lipogenesis (DNL), such as SCD inhibitors, target ChREBP directly. ChREBP also regulates hepatokines like FGF21, HGFAC, lipocalin 13, and SNOC1, which play roles in NAFLD pathogenesis; FGF21 analogues are currently in phase II trials, reviewed in [73]. TXNIP, another ChREBP target, is being studied for type 2 diabetes treatment. ChREBP indirectly affects bile acid metabolism through G6P-mediated regulation of CYP8B1, potentially influencing FXR agonist actions [166]. Metformin lowers glucose by inhibiting ChREBP’s transcriptional activity and downregulates targets, including G6PC, PKLR, and PCSK9, contributing to its cholesterol-lowering effect [167,168,169,170,171,172,173,174,175,176,177,178,179]. While ChREBP activation promotes hepatic lipid accumulation, it may protect against NASH events. Dietary carbohydrates stimulate ChREBP, while chronic high-fat and saturated fat intake reduces its activity. There are still unanswered questions regarding ChREBP, particularly the metabolic functions of ChREBPβ. A genetic mouse model lacking ChREBPβ recently published provided some insights, but more molecular research is necessary to determine if ChREBPβ operates as a transcriptional relay of ChREBPα in response to carbohydrates like fructose [119].

### 5.4. ChREBP Inhibitors

Research on ChREBP inhibitors has advanced rapidly over the past two years (2024–2026), focusing on cancer therapy, metabolic diseases, and novel screening platforms.

A study established a specialized pharmacological screening toolkit using a high-throughput assay in INS-1E β-cells. The researchers identified three putative covalent inhibitors and two non-covalent chemical scaffolds capable of directly blocking ChREBP-driven transcription at carbohydrate response element sites, creating new chemical baselines for therapeutic design [179,180]. Targeting Liver Cancer with the Small Molecule SBI-993 [180], a study, identified ChREBP as a potent oncogene driving hepatocellular carcinoma (HCC). Crucially, the researchers demonstrated that pharmacological inhibition of ChREBP using the small molecule inhibitor SBI-993 significantly suppressed in vivo HCC tumor growth and cell proliferation without causing systemic toxicity, identifying it as a viable clinical candidate [180].

Molecular Glues Stabilizing ChREBPα/14-3-3 Complexes: Instead of traditional competitive inhibition, this investigation details a novel approach using structure-based optimization to create “molecular glue” stabilizers. These small molecules selectively stabilize the interaction between ChREBPα and 14-3-3 proteins, prompting cytoplasmic retention and suppressing the toxic surge of ChREBPβ that causes pancreatic β-cell death during diabetes progression [181].

### 5.5. Therapeutic Trade-Offs of ChREBP Inhibition [179,180,181]—Translational Limitations

Following the considerations expressed so far, ChREBP inhibition has several potential benefits: reverses MASLD; blocks de novo lipogenesis, clearing excess hepatic lipids; improves insulin sensitivity; lowers lipid accumulation to restore whole-body glucose tolerance; suppresses hepatocarcinoma as it curbs the altered metabolic pathways that fuel tumor growth; protects beta cells; and prevents glucotoxicity-induced cell death by lowering oxidative stress.

On the other hand, and due to the duality of functions as well as the site-specific actions (notably the difference between hepatic and enterocyte actions), several potential risks and toxicity must be considered: severe fructose intolerance as it disrupts intestinal/hepatic processing, leading to lethal ATP depletion, accelerates liver fibrosis; removing ChREBP-α allows profibrogenic signals, worsening tissue scarring; adipose tissue dysfunction; total deletion in white fat triggers severe localized insulin resistance.

A strategic outlook on the future of this exciting research avenue will consider that blunt, systemic inhibition of ChREBP introduces severe toxicity. Modern drug development is shifting toward tissue-specific targeting or isoform-selective molecules to block the toxic ChREBP-β surge while preserving essential baseline glucose processing.

### 5.6. Monitoring Tools

Given the unique metabolic effects of fructose, evaluation should include parameters beyond fasting glucose.

Given the mechanisms discussed in Section 2, Section 3 and Section 4, beyond the stable isotopes and MRS research techniques previously mentioned [21], monitoring at the clinical level may take advantage of:

• Blood Biomarkers: Regularly assess serum uric acid (which may be elevated due to fructose as shown in Section 2), fasting insulin and HOMA-IR (early markers of insulin resistance), lipid profile (triglycerides and VLDL), liver enzymes (ALT, AST, and GGT), and HbA1c for comprehensive glycemic control.
• Imaging and Physical Markers: Utilize FibroScan technology to assess hepatic steatosis and fibrosis and monitor waist circumference as an indicator of visceral adiposity and recovery in metabolic health.

### 5.7. Conclusions

Current evidence underscores ChREBP as the central regulator of fructose-induced hepatic lipogenesis. Unlike glucose, which is subject to tight systemic regulation, fructose provides a rapid substrate influx that constitutively activates ChREBP, bypassing traditional metabolic checkpoints. This chronic activation not only fuels the synthesis of new FA but also impairs lipid oxidation, creating a “perfect storm” for the development of MASLD (contingent to the TG export capacity of the liver). In recent years, focus on the metabolic impact of fructose is no longer viewed solely through a hepatic lens; rather, it is defined by the collaborative ChREBP signaling between the intestine and the liver. While intestinal ChREBP acts as a primary barrier by facilitating fructose absorption and initial clearance, its saturation leads to a secondary hepatic overflow. ChREBP represents a pivotal yet complex target in the fight against fructose-mediated metabolic disorders. Normally, ChREBP helps prevent harmful buildup of glycolytic byproducts by promoting glycogen storage and glucose export. However, high sugar consumption can overwhelm these defenses, leading to excessive lipogenesis. While glucose strongly activates ChREBP in vitro, fructose has a more potent effect in vivo due to distinct metabolic pathways. We hypothesize that one of these mechanisms could be MG-induced stabilization of ChREBPα, prolonging its action by impairing ubiquitination. This is a speculative mechanism that needs further studies, and a workflow is suggested. Numerous other unresolved questions persist concerning ChREBP, especially in relation to the metabolic roles of ChREBPβ. A genetic mouse model lacking ChREBPβ provided some insights, but more molecular research is necessary to determine if ChREBPβ operates as a transcriptional relay of ChREBPα in response to fructose. Ultimately, future research should aim to clarify how—and which—glucose metabolites govern ChREBP activity in cells. The complex regulatory mechanisms governing ChREBP present considerable challenges in forecasting the outcomes of targeted interventions. While its role in promoting DNL makes it an attractive candidate for inhibition, its fundamental necessity for normal glucose sensing and transport presents a significant clinical challenge. The paradox lies in its dual function: maintaining metabolic flexibility under acute loads while driving insulin resistance under chronic fructose consumption. Moving forward, research must transition from identifying ChREBP’s downstream targets to uncovering the specific co-factors that differentiate its physiological benefits from their pathological consequences. Collectively, these arguments underscore the need for further investigation into human fructose metabolism and support the basis for public health guidelines advocating reduced sugar intake and the determination of safe consumption limits.

## Figures and Tables

**Table 1:** Comparison between ChREBP’s two isoforms.

Feature	ChREBP-α (Alpha)	ChREBP-β (Beta)
*Length*	Full length (852–864 amino acids)	Truncated N-terminus (687 amino acids)
*Transcription Start Site*	Alternative Exon 1a	Alternative Exon 1b
*Basal Tissue Expression*	Dominant and highly expressed	Extremely low baseline expression
*Glucose Responsiveness*	Highly dependent on glucose levels	Constitutively active
*Low-Glucose Inhibitory Domain (LID)*	Present (blocks activity in low glucose)	Absent (lacks first 177 amino acids)
*Nuclear Shuttling Signals (NLS/NES)*	Present	Absent
*Subcellular Localization*	Cytoplasmic at low glucose; moves to nucleus	Permanently nuclear
*Transcriptional Potency*	Basal/Moderately regulated	~20-fold more potent than alpha
*Dimerization and DNA Binding Domains*	Conserved C-terminus (bHLH/LZ)	Conserved C-terminus (bHLH/LZ)
*Primary Pathological Role*	Drives adaptive cell proliferation	Drives glucotoxicity and lipid buildup

## Data Availability

No new data were created or analyzed in this study. Data sharing is not applicable to this article.

## Abbreviation

ACC	Acetyl-CoA Carboxylase
ACLY	ATP-Citrate Lyase
ACSS2	Acyl-CoA Synthetase Short-Chain Family Member 2
AHA	American Heart Association
AMP	Adenosine Monophosphate
AMPK	AMP-Activated Protein Kinase
ATF6	Activating Transcription Factor 6
bHLH/LZ	Basic Helix–Loop–Helix–Leucine Zipper
ChoRE	Carbohydrate Response Element
ChREBP	Carbohydrate Response Element-Binding Protein
CRY1	Cryptochrome-1
DNL	De Novo Lipogenesis (the synthesis of fatty acids from acetyl-CoA)
DHAP	Dihydroxyacetone Phosphate
D-lactate	D-Lactate (distinct from L-lactate produced during glycolysis)
FFA	Free Fatty Acids
FGF21	Fibroblast Growth Factor 21
FASN	Fatty Acid Synthase
F1P	Fructose-1-Phosphate
F6P	Fructose-6-Phosphate
F26BP	Fructose-2,6-Bisphosphate
G6P	Glucose-6-Phosphate
GAP	Glyceraldehyde 3-Phosphate
GK	Glucokinase
GKRP	Glucokinase Regulatory Protein
GLUT2	Glucose Transporter 2
GLUT5	Glucose Transporter 5
GLUT8	Glucose Transporter 8
Glo1	Glyoxalase I
Glo2	Glyoxalase II
GRACE	Glucose-Response Activation Conserved Element
GSM	Glucose-Sensing Module
G6PC	Glucose-6-Phosphatase
G6PT1	Glucose-6-Phosphate Transporter 1
HGFAC	Hepatocyte Growth Factor Activator
HFCS	High-Fructose Corn Syrup
HFCS-55	High-Fructose Corn Syrup with 55% Fructose and 45% Glucose
IRS-1	Insulin Receptor Substrate 1
IRS-2	Insulin Receptor Substrate 2
KHKc	Ketohexokinase c (fructokinase)
LDL	Low-Density Lipoprotein
LID	Low-Glucose Inhibitory Domain
MASLD	Metabolic-Associated Steatotic Liver Disease
MetS	Metabolic Syndrome
MCR	Mondo Conserved Region
MG	Methylglyoxal
MLX	Max-Like Protein X
NADPH	Nicotinamide Adenine Dinucleotide Phosphate (reduced form)
NES1	Nuclear Export Signal 1
NES2	Nuclear Export Signal 2
NLS	Nuclear Localization Signal
NLRP3	NOD-LRR and Pyrin Domain-Containing Protein 3
Nrf2	Nuclear Factor-Erythroid Factor 2-Related Factor 2
OGT	O-GlcNAc Transferase (implied in O-GlcNAcylation)
PERK	Protein-Kinase RNA-Like Endoplasmic Reticulum Kinase
PPAR	Peroxisome Proliferator-Activated Receptor
PPK-2	Phospho-Fructokinase 2
SCD1	Stearoyl-CoA Desaturase 1
SCFA	Short-Chain Fatty Acid
SREBP-1	Sterol Regulatory Element-Binding Protein 1
SREBP1c	Sterol Regulatory Element-Binding Protein 1c
SSB	Sugar-Sweetened Beverage
TG	Triglyceride
TRL	Triglyceride-Rich Lipoproteins
TXNIP	Thioredoxin-Interacting Protein
UPR	Unfolded Protein Response
VLDL	Very-Low-Density Lipoprotein
